# Enhancing diagnostic and intervention-related skills with adaptive feedback: Study protocol for a simulation-based learning study on symptoms of learning and behavioural difficulties

**DOI:** 10.1371/journal.pone.0361425

**Published:** 2026-11-13

**Authors:** Frank Niklas, Stefan Ufer, Dilara Isik, Moritz Klippert, Luzia Hofer, Elisabeth Bauer, Michael Sailer

**Affiliations:** 1 Department of Psychology, University of Munich (LMU), Munich, Germany; 2 Mathematics Education, University of Munich (LMU), Munich, Germany; 3 Learning Analytics and Educational Data Mining, University of Augsburg, Augsburg, Germany; GrandEdu GmbH, GERMANY

## Abstract

**Background:**

This project uses simulated digital case scenarios to analyse diagnostic and intervention-related skills of teacher, school psychology, and medical students concerning primary school children’s difficulties in mathematics and their hyperactive, inattentive and impulsive behaviours. Here, the conditions for an effective use of macro- and micro-adaptive feedback – powered by natural language processing (NLP) methods – compared to static feedback will be the focus. The aim is to identify effective types of feedback for students who differ in their prior diagnostic and intervention-related knowledge.

**Methods/design:**

We compare three groups of university students (medical students, school psychology students, and pre-service teachers) with varying professional specialisations and corresponding knowledge bases as learning prerequisites using simulated digital case scenarios. In validation study 1a, we analyse students’ case processing and validate our measures for students’ prior knowledge. Furthermore, the results are used for informing the design and refinement of micro- and macro-adaptive feedback. Validation study 1b investigates static, macro-adaptive, and micro-adaptive feedback. In Study 2, by using “think aloud”-methods and trace data, we investigate to what extent learners benefit differently from the processing of digital case scenarios in the absence of any feedback and what kind of cognitive, metacognitive, motivational learning processes occur. In study 3, we investigate how the form of feedback in the digital case scenarios affects students’ acquisition of new diagnostic and intervention-related skills and how students process the different forms of feedback. Finally, Study 4 examines the extent to which different forms of feedback are helpful for students with low vs. high prior knowledge.

**Discussion:**

This project will analyse which benefits feedback with different degrees of adaptivity can provide for learning diagnostic reasoning and the planning of related interventions, especially when considering potential interaction effects with learners’ varying prior professional as well as diagnostic and intervention-related knowledge and diagnostic processing.

## Introduction

Children’s early mathematical and behavioural skills are not only important prerequisites for academic achievement in school but are also a critical platform for their successful functioning as individuals later in society [1]. Consequently, it is of great importance to identify children with learning and behavioural difficulties early to prevent negative academic trajectories by proposing and applying appropriate intervention measures for them. However, early identification of learning and behavioural difficulties is a challenge for professionals in contact with young children such as teachers, school psychologists, and physicians. Here, the training of professional skills (i.e., diagnostic- and intervention-related skills) might help to address this challenge. With our simulation-based training approach, we aim at supporting university students’ learning of these skills.

This project is part of the SHARP initiative (SHARP = Simulation-based learning in higher education: Advancing research on process diagnostics and personalised interventions). The overarching goal of the Collaborative Research Centre SHARP (DFG TRR 419/1–542251251) is to contribute to theory-building on personalising teaching and learning using simulations in higher education. Here, SHARP focuses on diagnosing and intervening as two knowledge-based professional practices that are considered highly relevant in many academic professions such as education and medicine [2].

### Diagnostic and intervention-related skills for learning and behavioural difficulties

The focus of this project within SHARP lies on the diagnosis of learning and behavioural difficulties [3–6] which are highly relevant for the practice of physicians (in particular psychiatrists) and school psychologists (often specialised teachers) who make official clinical diagnoses. However, teachers with no specific background in school psychology also are involved in (pedagogical) diagnosing by identifying signs of learning and behavioural difficulties, reacting adequately, and directing learners to external support, if necessary [7–9].

Teachers and physicians require both, specific knowledge about symptoms and related interventions, as well as more general diagnostic skills [10–12]. In the context of mathematical learning and behavioural difficulties, professionals should be able to identify and assess relevant symptoms such as impaired basic processing of numbers and quantities [3,4] and symptoms of inattention, hyperactivity, and impulsivity [5,6]. In practice, different professions (teachers, school psychologists, and physicians) must collaborate, share knowledge, and provide advice, oftentimes without any prior training to do so.

Specifically, as responsibilities are distributed across professionals from different fields, communicating considerations regarding potential diagnoses and interventions – that is, formulating meaningful and reasoned justifications for diagnostic conclusions – is a key subskill that requires specific training [10,13]. However, due to a lack of practical training opportunities during higher education, these diagnostic skills and knowledge about appropriate interventions are often acquired insufficiently [14]. Additionally, repeated practice of relevant skills is rarely possible. Moreover, students of the different professions (here, medical, teacher, and school psychology students) will differ in their prior general diagnostic and intervention-related knowledge, their specific knowledge on learning and behavioural difficulties in pupils, and their motivation to engage in this topic.

Following the SHARP framework [2], we assume that higher levels of diagnostic- and intervention-related skills are indicated by more accurate and better justified diagnoses and interventions, as well as by an increasing quality and efficiency of diagnostic and intervention-related activities, e.g., by applying profession-specific standards for generating and evaluating differential hypotheses [10,13,15] or for treating a certain type of learning or behavioural difficulty. Similarly, the quality of the diagnostic process (leading to this diagnosis) explains aspects of the accuracy and justification of the diagnosis even beyond professional knowledge [16]. Independent of their study programme, and thus their different learning prerequisites, all students are expected to accurately diagnose learning and behavioural difficulties in pupils, in the sense of identifying whether learning or behavioural difficulties are within age-typical variation, or whether they indicate potentially clinical difficulties. Further, they are expected to identify next steps and, thus, to recommend potential intervention measures.

### Simulation-based learning for enhancing diagnostic skills in higher education

Simulations can be a solution to train the necessary diagnostic- and intervention-related skills as they enable learners to repeatedly engage in the learning in complex situations and to balance authenticity of learning experiences and cognitive demand [17,18]. Further, incorrect solutions do not have detrimental consequences and, on the contrary, learners may even profit from their errors [19]. Empirical studies and meta-analyses have highlighted the effectiveness of simulation-based learning in various educational contexts [20]. However, simulation-based learning also requires instructional support to exploit its effectiveness [20–23].

In this context, especially process-oriented feedback that elaborates on diagnostic and intervention-related activities offers high potential for learning [24–26]. For instance, feedback can be static in the form of an expert solution that presents an effective task processing to the learner [27], it can be adapted on a macro-level (i.e., a specific expert solution adapted to typical mistakes of a certain group of learners such as medical students, teachers, or school psychologists or it can be adapted on a micro-level [2,28,29], that is, considering individual learners’ current performance to provide relevant information for facilitating their reasoning [30,31].

To capture learners’ diagnostic or intervention-related reasoning, especially in the form of their justifications, open answer questions in which students submit written responses are a suitable approach [10,13]. However, for instructors in higher education, providing feedback on open answer questions for simulated cases is time-consuming [32]. Consequently, ready-made feedback could be used instead. Here, an expert justification and written response can be seen as static feedback without any adaptivity. Further, feedback may be adapted on a macro-level according to the background of the learner and typical mistakes of such learners. Although such feedback is more adaptive than the static feedback, it still does not provide adaptivity on an individual level.

Here, harnessing the increasing potential of artificial intelligence, specifically natural language processing [28,33], for an automation of real-time adaptive feedback on students’ diagnostic justifications in simulated digital case scenarios can be a time-efficient approach for enhancing learning in higher education. In recent years, NLP has been increasingly adopted for automatic assessment in digital learning environments such as simulations [34–36]. For instance, recent advances in NLP – namely, large language models (e.g., BERT, GPT) – can offer parsing complex written responses and generating elaborated micro-adaptive feedback [37,38].

### Feedback and learners’ diagnostic and intervention-related skills

Prior research has shown positive effects of micro-adaptive feedback which was based on learners’ current performance (i.e., a written text) and provided in real-time, on the quality of reasoning in complex tasks [28,38–40]. Such feedback can support learners’ knowledge acquisition (diagnostic and intervention-related skills) and metacognitive engagement (self-regulation) in the further learning process, enabling a more accurate assessment of the own learning status (metacognitive monitoring) and a more precise regulation of learning behaviour (e.g., adjustment of the invested effort and learning time) than less effective adaptation of learning experiences by the learners themselves [41]. Here, adaptive feedback could provide better support due to its higher specificity than the more generic counterpart of static feedback; the effectiveness of feedback, however, is influenced by several moderators such as prior learner skills and task difficulty [25,42,43].

The specific learning mechanisms of macro- and micro-adaptive feedback caused by the higher specificity and fit of the feedback information to learners’ needs in simulations are still relatively unexplored and might range from reducing cognitive resource depletion to facilitating self-regulation and increasing learners’ motivation [39]. When investigating these potential mechanisms, learners’ type and amount of task-related prior knowledge may play a key role as prior knowledge facilitates task understanding, making learning tasks less cognitively demanding [44], easier to monitor [45], as well as more rewarding [46]. Therefore, different feedback types may be processed differently by different learner groups. For instance, learners with low prior professional knowledge and diagnostic and intervention-related skills may profit more from very specific micro-adaptive feedback, while learners with more prior knowledge and skills might process macro- and micro-adaptive feedback equally well, as they have the spare cognitive resources to process less specific feedback (i.e., macro-adaptive feedback).

In a systematic review, Fleckenstein et al. [47] identified only a few well-controlled studies on the learning effectiveness of adaptive feedback, as studies often lack adequate implementation of adaptive feedback. In addition, the effectiveness of adaptive feedback employing transformer models based on large language models is an important research desideratum, as these algorithms offer high efficiency and flexibility, but are yet to be systematically harnessed and researched – especially, as initial studies indicate that their feedback can offer positive effects, but is not always well received by the learners [40,48–52].

### Rationale for the study, study aims, and hypotheses

This project will analyse which benefits different forms of adaptive feedback can provide for learning diagnostic reasoning and the planning of related interventions, especially when considering potential interaction effects with learners’ varying prior professional knowledge and diagnostic and intervention-related skills. Realistic simulated case scenarios of pupils with behavioural and learning difficulties will be applied to train medical, teacher, and school psychology students’ diagnostic and intervention-related skills, supported by the provision of effective text-based feedback in-between the processing of the simulated cases. Here, we expect different prior knowledge and diagnostic and intervention-related skills across the three groups despite substantial within-group variance. Consequently, this project focuses on the interaction between students’ prior professional knowledge, their diagnostic, and intervention-related skills and different forms of feedback (static/macro-adaptive/micro-adaptive) with the goal to enhance students’ diagnostic and intervention-related skills via simulation-based learning.

The identification of learning and behavioural difficulties is of great importance for physicians, teachers, and school psychologists. Here, the primary goal is to test which kind of feedback better supports students’ knowledge and skills acquisition depending on variations in their prior knowledge. Our project builds on the SHARP framework [2] for conceptualising the diagnostic and intervention activities, as well as the cognitive, metacognitive, motivational, and social learning processes involved in case processing. Further, the project builds on the application of adaptive feedback and aims to contribute to theory-building for the interaction of feedback and students’ prior knowledge in the context of diagnostic and intervention-related skills concerning learning and behavioural difficulties. The following hypotheses will be tested in this project:

Hypothesis 1.1: Teacher students will have less prior professional knowledge and diagnostic and intervention-related skills for the processing of simulated digital case scenarios of pupils with behavioural difficulties compared to medical and school psychology students (Validation Study 1a).

Hypothesis 1.2: Medical students have less prior professional knowledge about and less interest in mathematical learning difficulties compared to teacher and school psychology students (Validation Study 1a).

Hypothesis 1.3: Macro- und micro-adaptive compared to static feedback will better facilitate diagnostic and intervention-related decisions and justifications during the processing of simulated digital case scenarios of pupils with learning and behavioural difficulties (Validation Study 1b).

Hypothesis 2.1: Participants with more prior professional knowledge and diagnostic and intervention-related skills will show better cognitive, metacognitive, and motivational learning processes (Study 2).

Hypothesis 2.2: Participants with more prior professional knowledge will show better diagnostic and intervention-related skills (Study 2).

Hypothesis 3.1: Receiving macro-adaptive feedback will enhance diagnostic and intervention-related skills more compared to receiving static feedback (Study 3).

Hypothesis 3.2: Receiving micro-adaptive feedback will enhance diagnostic and intervention-related skills more compared to receiving macro-adaptive feedback (Study 3).

Hypothesis 4.1: Participants with less prior professional knowledge and diagnostic and intervention-related skills will benefit more from micro-adaptive feedback than from macro-adaptive and static feedback (Study 4).

Hypothesis 4.2: Participants with more prior professional knowledge and diagnostic and intervention-related skills will profit more from macro- and micro-adaptive feedback than from static feedback (Study 4).

## Materials and methods

Our project comprises two validation studies (1a, b) and three main studies (2–4) that focus on analysing and fostering diagnostic and intervention-related skills of university students in the context of mathematical and behavioural difficulties in pupils. Simulated digital case scenarios will be applied to enhance these skills, while considering the students’ varying prior professional knowledge and the type of feedback they receive while working with the case scenarios. We will analyse in which way students with a different study subject differ concerning their cognitive prerequisites, their case processing, and whether different types of feedback result in different learning effects.

Further, we will use think aloud-measures [53] and trace data to analyse the learning processes of learners with varying prior knowledge. We will also analyse the support different feedback (static/macro-adaptive/micro-adaptive) has on participants’ diagnostic and intervention-related skills and learning processes. Finally, we investigate whether a personalisation of feedback, based on prior knowledge, will lead to better diagnostic and intervention-related skills. Recruitment for validation study 1a will take place from April to July 2026 and data collection will be completed in August 2026. First results of study 1a can be expected towards the end of 2026. The subsequent studies will take place between the end of 2026 (study 1b) and summer 2028 (study 4).

### Study overview

Table 1 provides a general project overview about the validation and main studies.

**Table 1:** Overview of the study.

Study	1a	1b	2	3	4
**Description**	Validation study for the case scenarios and the assessment tools; data for adaptive feedback realizations	Validation study for the optimization of the case scenarios and adaptive feedback realizations	Correlational study on learning prerequisites and learning processes	Experimental study on the effect of different forms of feedback	Experimental study on the interaction of different forms of feedback with different learning prerequisites
**Sample**	N = 111	N = 111	N = 135	N = 180	N = 228
**Design**	2 group comparison	2 group comparison	Regression	3 group comparison	2x3
**Power** **analyses**	Mean comparisons, 3 groups (f = .55), α = .05, power = .90; N = 108	Mean comparison, 3 groups (f = .55), α = .05, power = .90; N = 108	Slope H_0_/H_1_ = 0/.25, α = .05, power = .90; N = 130	Group comparisons (f = .15); α =.05, power =.95; N = 177	Group comparisons (f = .15); α = .05, power = .95; N = 228
**Independent Variables**	Students’ subject (T vs. M & P; M vs. T & P)	Static vs. macro-adaptive & micro-adaptive feedback	Cognitive learning prerequisites	Static, macro-adaptive, micro-adaptive feedback	Learning prerequisites & static, macro-adaptive, micro-adaptive feedback

In validation study 1a, baseline assessments will be conducted with the students, before they work on eight case scenarios as outlined below (see “simulated cases”). The case processing by the participants serves as a basis for the design of the adaptive feedback. Therefore, participants’ written answers will be analysed and coded. In addition, metacognitive learning processes will be assessed. In a first session, participants complete the pre-test, followed by 4 simulated pupil cases including diagnostic and intervention-related assessments after each case. The second session at a subsequent day will consist of 4 more pupil cases and a post-test.

Validation study 1b will follow the same procedure and investigates static, macro-adaptive, and micro-adaptive feedback (static: expert solution for diagnostic/intervention-related justifications; macro-adaptive: feedback concerning typical mistakes and knowledge deficits of the specific group of learners (i.e., medical, teacher, and school psychology students); micro-adaptive: written justifications are evaluated using a large language model (LLM) to provide adaptive feedback).

Study 2 will follow the same procedure as the validation studies and will include the same measures, but no feedback. Cognitive, metacognitive and motivational prerequisites will be assessed and cognitive, metacognitive and motivational learning processes during the processing of the simulated digital case scenarios will be analysed. Think aloud-methods and trace data will be additionally used during the processing of the second and third case scenarios. Participants’ reasoning about the case, their monitoring of the case processing, the case aspects they are most interested in, and the order in which this information is accessed will be assessed, automatically transcribed, coded, and analysed. These data will provide valuable information on participants’ cognitive, metacognitive and motivational learning processes to inform the feedback provided in studies 3 and 4.

Study 3 will follow the same procedure as the validation study 1b, comparing the three different forms of feedback, but will include two more case scenarios. Here, the focus will lie on the question how the different forms of feedback can improve participants’ diagnostic and intervention-related skills. After the baseline assessments, participants will initially work on three example case scenarios. Then, they work on four more case scenarios for which they receive either static, macro-adaptive or micro-adaptive feedback. Finally, they will work on three more case scenarios to test their diagnostic and intervention-related skills after receiving feedback and, lastly, will complete the post-tests.

Study 4 will follow the same procedure as Study 3 and include the different forms of feedback which will be optimised using the data from the previous studies 1a, 1b, and 3. In study 4, the feedback will depend on participants’ learning prerequisites and their performance in the pre-test case scenario processing. The aim is to develop a learner model to predict the potentially most beneficial type of feedback for each participant’s learning prerequisites.

### Samples

The samples in each of the studies will consist of three different groups of German university students (see also Table 1 for sample sizes). One third will be medical students, one third will be training to become primary school teachers which includes studying mathematics didactics. The last third will be students of school psychology, which means in the Bavarian context, in which this study is conducted, that these students are also training to become teachers, however, their studies follow a specific curriculum with a strong focus on (school) psychology and diagnostic skills. Participants are eligible for inclusion only if they belong to the relevant study groups for the respective study, individuals outside these groups are not recruited. For the intervention studies, only participants with complete study participation will be included in the analysis. Reasons for discontinuation or withdrawal from the study will be documented for all participants who do not complete the study. Participation will be voluntary and all participants will receive remuneration for their time. Recruitment for Study 1a started on April, 8^th^ 2026.

### Measures

Assessments in our project include participant surveys, test assessments, think-aloud measures, and the processing of the simulated cases including diagnostic- and intervention-related responses (for an overview, see Table 2). Within the SHARP context, the various projects conducted will include the same measures for comparison and meta-analytic approaches. In addition, additional assessments will be conducted in each project which will focus on specific aspects, that are key for each individual project.

**Table 2:** Overview of the study variables.

Domain	Concept	Test instrument	Timing of assessment
Learning prerequisites			
Meta-cognitive	Learning strategies	LIST-K (selected from Boerner et al.; Klingsieck; Wild & Schiefele [57–59])	Pre-test
Social	General social skills	Social skill questionnaire (selected and adapted from Brandl et al. [16], based on Hesse et al.; Liu et al. [60,61])	Pre-test
Motivational-affective	Interest	Interest scale [62]	Pre-test
Motivational-affective	Self-concept in diagnosing and intervening	Diagnosing & intervening self-concept scale (designed by the Collaborative Research Centre SHARP)	Pre-test
Cognitive	Prior knowledge about children’s learning and behavioural difficulties	Prior knowledge test (developed by the authors, based on Bichler er al.; Kron et al.; Sailer et al.; Wehren-Müller [11,21,39,63])	Pre-test
Cognitive	Speeded deductive reasoning/ Intelligence-screening	mini-q [64]	Post-test

Experiences of the learning process and behaviour			
Cognitive	Cognitive load	Klepsch scale (second version of Klepsch et al. [65], as used in Stadler et al. [66])	Interim- & post-test
Motivational-affective	Situational satisfaction of psychological needs	Situational psychological need satisfaction scale (adapted from Sailer et al. [67], based on Ryan and Wilde et al. [68,69])	Interim- & post-test
Meta-cognitive	Situational strategies	Situational LIST-K (selected and rewritten based on Boerner et al.; Klingsieck; Wild & Schiefele [57–59])	Interim- & post-test
Motivational-affective	Epistemically-related emotions	Epistemically-related emotion scale (EES short scale, selected from Pekrun et al. [70])	Interim- & post-test
Motivational-affective	Values (intrinsic, attainment, utility), costs	Gaspard scale (selected and strongly adapted based on Gaspard et al. [71])	Interim- & post-test
Motivational-affective	Self-efficacy	Self-efficacy scale (selected and adapted from Holzberger et al. [72], based on Schwarzer and Jerusalem [73])	Interim- & post-test
Perception of the Learning Environment			
Scaffolding and personalization	Perceived Scaffolding, adaptivity, adaptability, quality of personalisation	Perceived scaffolding and personalization scale (designed by the Collaborative Research Centre SHARP)	Post-test
Authenticity	Perceived authenticity	Authenticity scale (Chernikova et al. [74], based on Schubert et al. and Seidel et al. [75,76])	Interim- & post-test
Involvement/presence	Involvement	Involvement scale (Chernikova et al. [74], based on Frank; Seidel et al.; Vorderer et al. [76–78])	Interim- & post-test
Task difficulty	Perceived task difficulty and effort	Wirth scale (selected from Wirth et al. [79])	Interim- & post-test
Subjective Learning Outcomes			
Subjective performance	Subjective performance and learning success	Subjective performance scale (designed by the Collaborative Research Centre SHARP)	Interim- & post-test
Learning satisfaction	Outcome satisfaction and perceived learning	Alqurashi scale [80]	Interim- & post-test

*Note.* Timing of the assessment: Pre-tests are assessed before case screening at the first measurement point; Interim-tests are conducted after processing the simulated digital case scenarios at the first measurement point; Post-tests are conducted at the end of the second measurement point.

### Demographic measures

Participants age, gender, their study subject and prior years of studying and the final mark in their university-entrance diploma acquired at a secondary school in Germany will be assessed. Further, participants’ cognitive, meta-cognitive, motivational, and social learning prerequisites, learning process data and learning outcomes will be surveyed. Learning prerequisites comprise learning strategies, social skills, interest, and self-concept. Learning process data consist of participants’ cognitive load, situational strategies, epistemic emotions, value and costs appraisals, and their self-efficacy beliefs. Moreover, participants are asked to provide insight into their perception of the learning environment concerning scaffolding, adaptivity, adaptability, personalization quality, authenticity, involvement, and task difficulty. Finally, the outcome variables include subjective performance and outcome satisfaction. All these variables comprise Likert-type questionnaire items (see Table 2 below).

### Test instruments

Participants prior knowledge about behavioural and learning difficulties will be assessed via multiple choice tests. A short test of basic cognitive abilities (mini-q) is used to assess basic cognitive abilities of the participants during the post-test. In each of the project studies, survey assessments concerning participants learning prerequisites, the learning processes and their experience of the learning environment will be conducted as pre-, interim- or post-tests (see Table 2).

### The simulated cases

After the baseline assessments took place, the participants will be introduced to our simulated cases.

### Content and case overview

The content of these cases comprises eight (ten in study 3 and 4) digital, simulated case scenarios depicting grade two pupils exhibiting varying symptoms of behavioural and learning difficulties (for an overview, see Table 3). The cases are constructed to represent a broad range of pupil profiles, ranging from little observable to severe difficulties and various combinations of these difficulties. The cases begin with a standardized initial problem scenario that is identical across all pupils. Participants are informed that they have been consulted as an external professional, because a class teacher observed some difficulties in one of her students.

**Table 3:** Overview of simulated case scenarios.

Case name	Description
Child A	Severe attention deficit hyperactivity disorder, combined presentation (hyperactive-impulsive and inattentive presentation) & severe developmental learning disorder with impairment in mathematics (mathematical difficulties)
Child B	Severe developmental learning disorder with impairment in mathematics (mathematical difficulties)
Child C	Severe attention deficit hyperactivity disorder (predominantly hyperactive-impulsive presentation), slightly impairing academic functioning, with the exception of mathematics
Child D	Average student with mild mathematical difficulties
Child E	General learning difficulty and adaptive behaviour
Child F	Very good student with no learning or behavioural difficulties (gifted child)
Child G	Severe attention deficit hyperactivity disorder (predominantly inattentive presentation)
Child H	Severe language impairment due to language disorder in combination with learning German as a second language, consequently slightly impairing overall academic functioning
Child I	Mild developmental learning disorder with impairment in mathematics (mathematical difficulties) & mild attention deficit hyperactivity disorder, combined presentation (hyperactive-impulsive and inattentive presentation)
Child J	Severe oppositional defiant disorder & severe attention deficit hyperactivity disorder, combined presentation (hyperactive-impulsive and inattentive presentation), with high intellectual ability

After reading the initial problem scenario, participants are provided with access to multiple information sources that are identical across all cases. These sources include (1) reports (contributions from the music teacher, class teacher, the pupils themselves, and the pupil’s parents); (2) behavioural observations (in the school playground and in mathematics class); (3) work examples from the core primary school subjects in Germany (mathematics, German, and science and social studies); (4) additional art and sports documents (art projects and a sports event participation certificate); and (5) progress reports for grades 1 and 2, which in Germany are typically completed by the teacher and discussed in structured meetings with the pupils themselves, while their parents attend as observers (in the progress reports, information is available on the domains of social behaviour, learning behaviour, German, mathematics, science and social studies, and music).

Participants can access available information in any sequence. They are required to selectively identify and consult information that they consider relevant due to time constraints. The information varies in relevance to the assessment. Distributed across the varying informational source, participants can find relevant information on the pupils relating to their academic achievement in the core subjects of primary school, linguistic and mathematical skills, as well as their social and learning behaviour in and out of school.

One example is the case of a fictive grade two pupil, who is showing severe symptoms of an attention deficit hyperactivity disorder and a developmental learning disorder with impairment in mathematics (i.e., mathematical difficulties) [54,55]. These difficulties are reflected in multiple sources of information about the child. For example, reports and observations indicate that he has difficulty remaining quiet and seated, is easily distracted, and struggles to follow rules of conversation and play. The mathematics work samples further reveal substantial deficits in foundational mathematical skills. Specifically, they indicate a limited understanding of the place-value system and magnitude representation of numbers. In addition, he relies exclusively on counting strategies to solve arithmetic tasks. A more detailed case description of this child is provided in the S1 File. Electronic Supplemental. Case description.

### Technical implementation

The cases will be implemented in the CASUS online learning environment [56]. The simulated cases are structured according to CASUS’ card-based logic, in which information is distributed across interconnected cards (i.e., pages). These pages show information like students’ reports, behavioural observations, worksheet examples and additional documents (see case description).

Learners can navigate through the case using nonlinear paths. Learners can access information via buttons, which enables learners to actively decide what to access next, thereby simulating authentic decision-making processes in the diagnostic context. Moreover, the buttons have a mouse-over function to clarify the content that learners can expect, if they navigate to a specific page. We consider this important, because it helps learners make intentional decisions in CASUS during their diagnostic processing. We also aim to use the logfiles generated by learners during navigation and decision-making in our cases, enabling us to analyse their individual processing of the simulated digital case scenarios.

The cases integrate multimodal materials, including written descriptions, scanned worksheets, audio recordings of answers in a conversation, recordings of reading performance and short video sequences of classroom and playground behaviour. The technical implementation prioritises the use of multimodal and illustrative materials to create authentic and realistic cases that mirror diagnostic practice.

At any point during the case overview, the learners can navigate to an open-text response field containing structured prompts, where they are required to note down their diagnostic conclusions and planned interventions. The text field also saves answers when learners navigate back to other parts of the case. To complete a case, participants finalise their written case report based on their notes and the case material. This text data constitutes the primary output of learners’ case processing. Figs 1–4 provide screenshots of the platform and the information, that can be accessed by the learners. Please note that some of the content used in our case scenarios was developed with the support of artificial intelligence.

**Fig 1:**
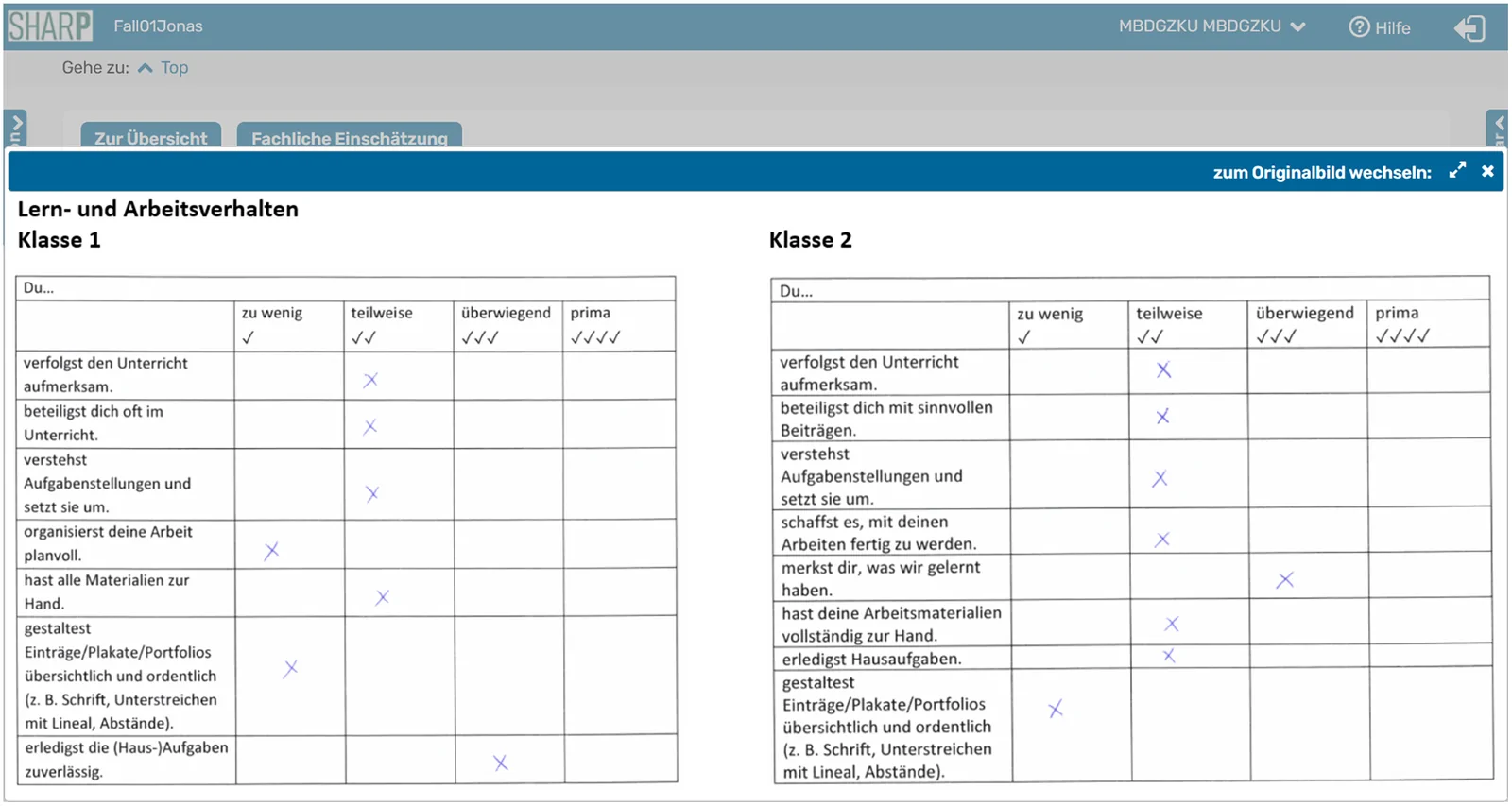
Example for progress reports for grades 1 and 2 with information on the child’s learning and working behaviour. On the left side information for grade 1 and on the right side for grade 2 is provided. Items include attention and participation during lessons and (home) work behaviour.

**Fig 2:**
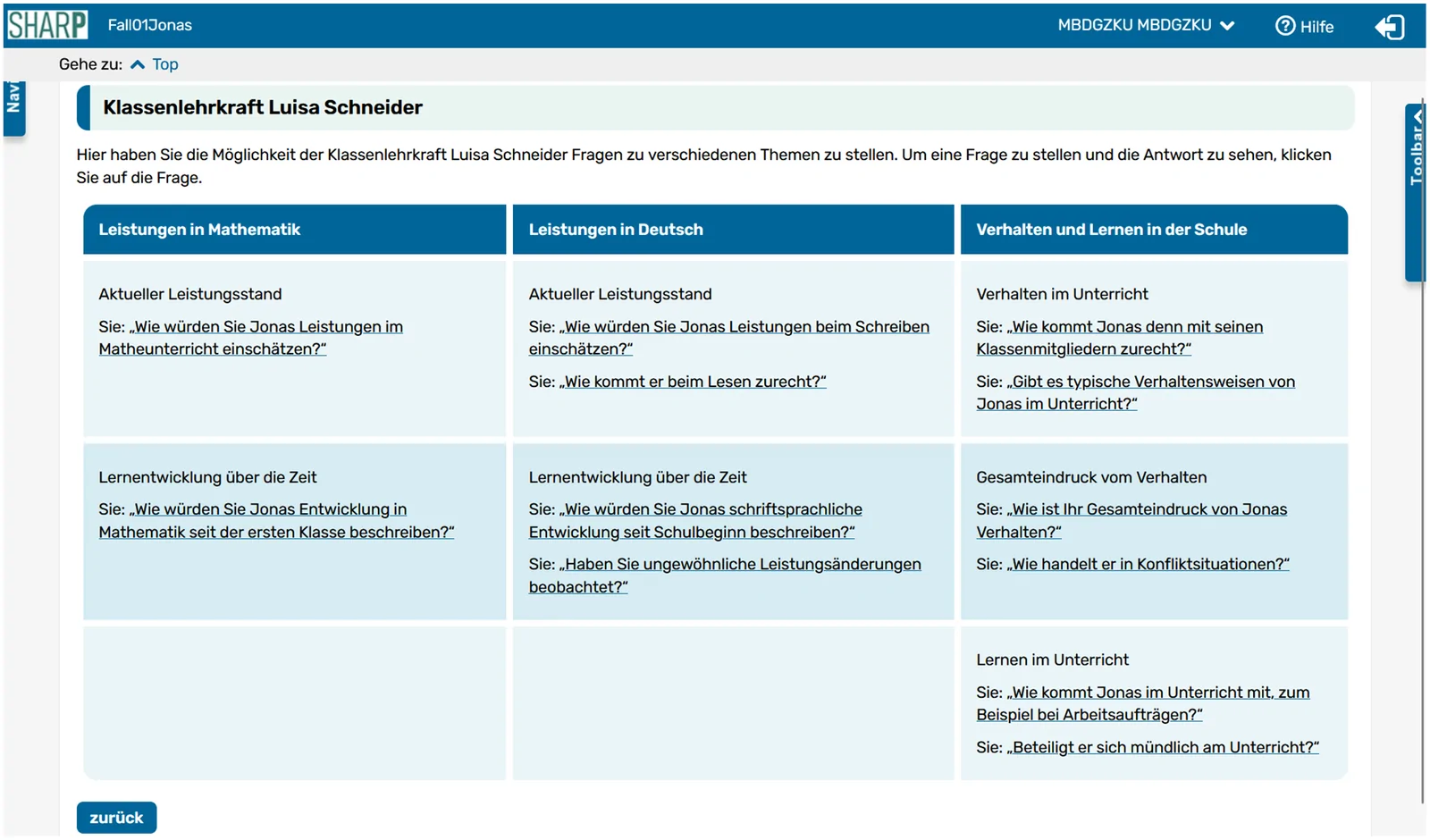
Example for a report by the class teacher. Study participants have the choice to ask specific questions to the class teacher of the child (fictive person Luisa Schneider) on the child’s performance in mathematics and German (current performance and learning trajectories), and about behaviour and learning in school. Short replies are provided when the participants click on a question.

**Fig 3:**
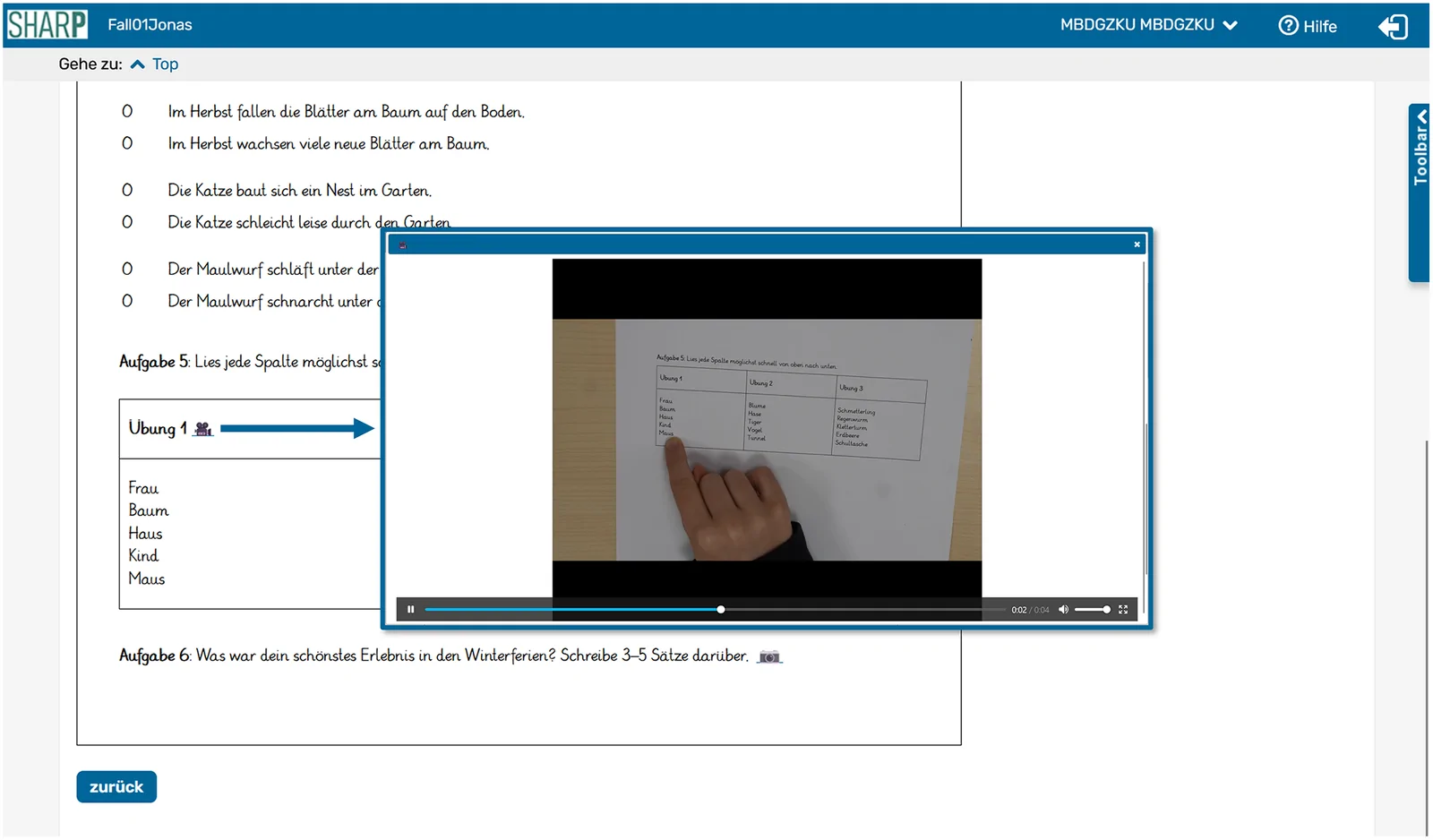
Worksheet example for German. Work example for the subject German. The participants are provided with a filled-in worksheet and can check the simulated child’s answers. In addition, they are provided with a reading example which is shown in three very short video clips.

**Fig 4:**
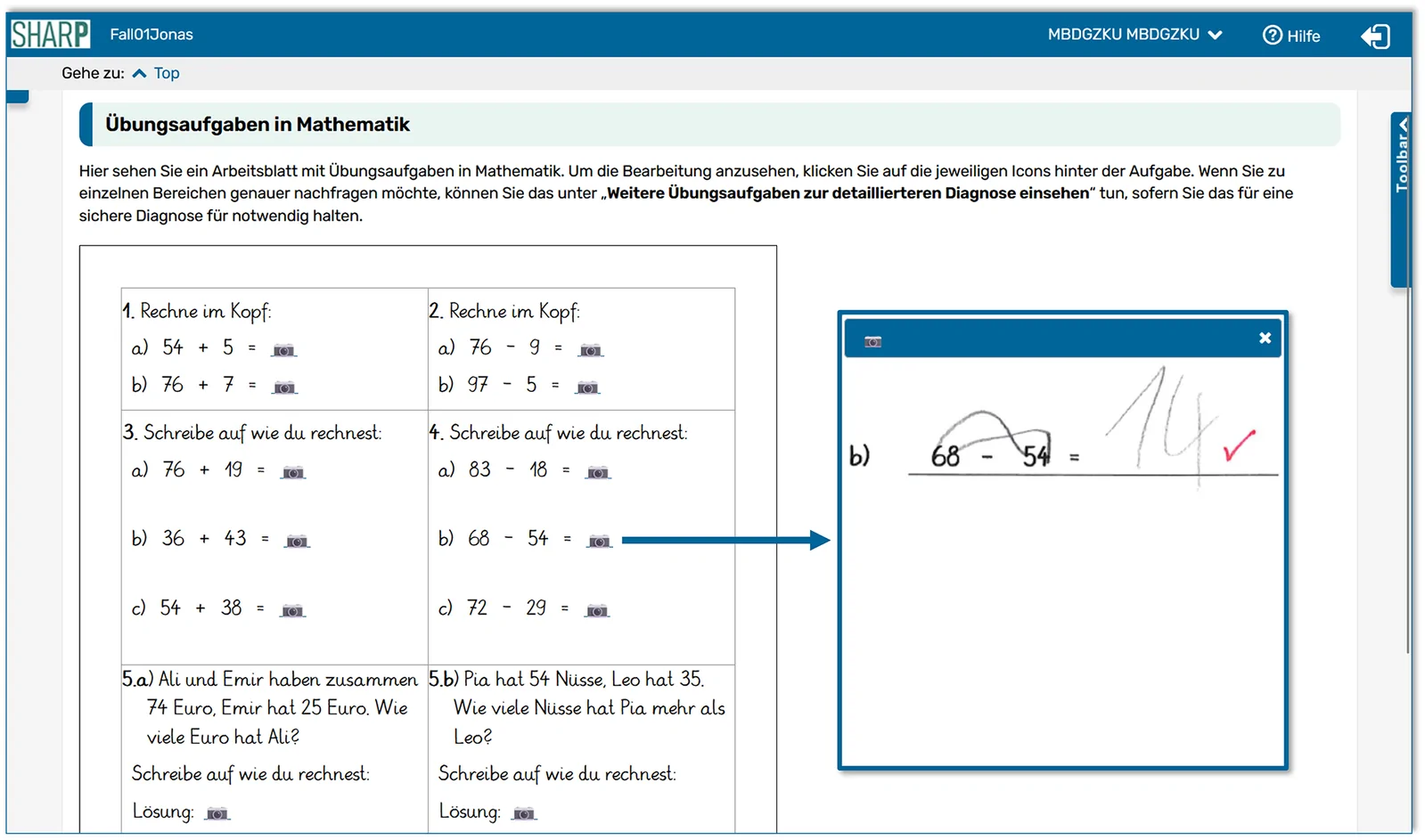
Worksheet example for Mathematics. Work example for the subject mathematics. The participants are provided with a filled-in worksheet and can check the simulated child’s answers. In addition, they are provided with a counting example which is show in short video clips.

### Feedback implementation

In Study 1b, 3 and 4, feedback is implemented and the systematic variation in the feedback (static/macro-adaptive/micro-adaptive) represents the core experimental manipulation of the project [28]. The empirical investigation focuses on the effectiveness of these different forms of feedback. Learners’ written case reports form the basis of the feedback intervention.

1) Static feedback offers general guidance on key diagnostic elements and principles for planning interventions, without referencing the learners’ written diagnosis in any way. Static feedback will be presented in the form of an expert solution, which was developed beforehand for every case and will be presented to the participants after their diagnostic report is submitted.
2) Macro-adaptive feedback addresses the learners’ written report and varies depending on the learners’ subject (i.e., medical, teacher, and school psychologist students). The feedback includes references to typical solutions and errors concerning diagnostic procedures and intervention planning strategies observed in these three groups of students. This results in three different versions of feedback being presented to learners depending on their study subject which they stated at the beginning of the study.
3) Micro-adaptive feedback responds to more specific aspects of the learners’ submitted report, such as the completeness of their evidence integration, the plausibility of their hypotheses as supported by case evidence and the coherence between their diagnostic conclusions and proposed interventions. To generate this feedback, an API-based integration with a large language model (LLM; specifically, gpt-oss-120b, hosted locally on a university server) is used after learners submit their responses. The LLM is implemented with a standardised system prompt. The prompt includes instruction on the expected feedback content and structure, including a few-shot component that comprises a small number of feedback examples. In addition, to integrate sufficient information on the case content and adequate processing, the case-specific expert solution (similar to the static feedback) is provided as a reference material. Considering the rapid pace of technological developments in this field, the model may be replaced with a more up-to-date version in future iterations of the platform. The LLM is prompted to compare the learner’s report with this expert solution and to address specific parts of their written statements such as hypotheses, evidence, and conclusions, including interventions. The quality of the generated feedback will be established through testing, prior to data collection, whereby LLM outputs will be evaluated by subject-matter experts for accuracy, responsiveness to the individual learner’s submission, and alignment with the expert solution. From the learners’ perspective, the feedback appears as an integral component of the case environment and is seamlessly connected to their previous case processing.

These distinctions result in different levels of personalisation for learners across the feedback formats (static/macro-adaptive/micro-adaptive). In every feedback condition, learners receive feedback directly within the learning environment after submitting their diagnosis on the respective page.

### Analytic strategy

The analytical plan includes reliability tests for our measures.For the validation studies, differences in prior knowledge and diagnostic- and intervention-related skills between the three student groups (teacher students, medical students, and school psychology students) will be examined using a one-way analysis of variance (ANOVA), with planned contrast analyses specified a priori to compare teacher students against medical and school psychology students, and medical students against teacher and school psychology students, according to hypotheses 1.1 and 1.2. Further, a one-way ANOVA with planned contrast analyses will be used to compare the three feedback conditions (static, macro-adaptive, and micro-adaptive) regarding the diagnostic- and intervention-related skills (hypothesis 1.3). In addition, regression analytic approaches and repeated measures analyses of variance will be conducted to answer the hypotheses in studies 2, 3, and 4. Missing data within completed responses is minimised by the laboratory-based study setting, in which all questions are mandatory to complete before proceeding. However, this does not preclude participants from discontinuing their participation entirely, in which case the dropout will be documented and the participant’s data will be excluded.

## Discussion

One important challenge for practice and research is to find ways to support future school psychologists, teachers, and physicians to being able to identify specific learning and behavioural difficulties in primary school children in order to also identify measures and next steps in attenuating these difficulties [3,7]. This project exploits new technological possibilities afforded by simulated learning platforms [56] and artificial intelligence [37] to teach and train different groups of university students in this task. Consequently, the long-term goal will be, that learners trained with our simulated cases will be better able to identify children with specific learning and behavioural difficulties in their later professions and that they will be better equipped to support these children either directly or by guiding them and their parents to adequate professional support.

Altogether, this study will lead to an empirically tested learning platform with simulated cases of primary school children.

### Conclusions and potential impact

We believe that this project will have a positive impact on several important areas:

(1) the diagnostic and intervention-related teaching in university education, that can readily be up-scaled as the simulated cases can be shared and used across interested university teachers
(2) the diagnostic and intervention-related skills in university students who work with these simulated digital case scenarios and receive support via appropriate feedback
(3) the development of applications which use specific large language models to provide feedback and that are trained to support university students’ learning in a meaningful way
(4) our knowledge about learner-treatment-interaction and thus about which kind of feedback is most helpful for which learner with which specific prerequisites.

Consequently, we believe that this project has the potential to not only provide us with important scientific insight, but also to have a positive impact on university students’ understanding of behavioural and learning difficulties in young children, their application of this knowledge, the learning and academic trajectories of these children, and thus in the long term also on the broader society.

## Supporting information

S1 File Electronic Supplement. Case description.
(DOCX)
