# Supplementary material for: Enhancing diagnostic and intervention-related skills with adaptive feedback: Study protocol for a simulation-based learning study on symptoms of learning and behavioural difficulties
Source: PLoS One. 2026 Nov 13;21(11):e0361425. doi: 10.1371/journal.pone.0361425 (PMC13766221; doi:10.1371/journal.pone.0361425)
Supplement: Supplementary file 1 [file pone.0361425.s001.docx]

**S1. Electronic Supplemental. Case description.**

One example is the case of a fictive grade two pupil, who is showing severe symptoms of an attention deficit hyperactivity disorder and a developmental learning disorder with impairment in mathematics (i.e., mathematical difficulties) [54,55]. Specifically, Child A exhibits high levels of hyperactive-impulsive and inattentive behaviour (see Table S1). Reports and observations of his behaviour reveal that he finds it difficult to remain quiet and seated and to follow rules of conversation (e.g., blurting out answers in class) and play (e.g., difficulties in waiting during games) in and out of class. He is said to act without consideration of the consequences and to have a rather low frustration tolerance, but also to calm down quickly and show insight after conflicts. Child A’s music teacher reports that he frequently arrives late.

These characteristics also impair his social functioning, and despite usually being friendly towards his classmates, he is perceived as exhausting and annoying by them. His difficulty to sustain attention becomes apparent in his distractibility, as demonstrated by his tendency to make careless errors or to leave unfinished sentences in his work examples. Moreover, Child A’s class teacher reports that he struggles to complete tasks that are rather unstructured.

According to his progress reports, Child A often forgets to bring all his school utensils to school. He also switches games in the middle of playing or distracts himself in mathematics class instead of solving the required task, as shown in the behavioural observations. Similarly, his parents explain that he easily loses interest in his homework, starting to do something else when he is not under close supervision.

In mathematics, class teacher reports indicate that he appears uninterested and reluctant to participate, although there have been some exceptions. For example, he was very engaged when the class learnt about geometry. Child A’s father also reports difficulties in mathematics without going into detail. The mathematics work samples – particularly two short videos showing Child A solving two arithmetic problems – indicate that he relies exclusively on counting strategies. Moreover, in both addition and subtraction, he consistently exhibits the ±1 errors typical of counting strategies. This error pattern is consistently observable across all solutions on the provided worksheet. At the same time, Child A demonstrates accurate and fluent counting abilities.

Additionally, Child A demonstrates only a superficial understanding of the place-value system. He can decompose two-digit numbers into tens and ones, but only at a procedural level. This allows him to solve addition and subtraction problems by counting the tens and ones separately. However, he shows no understanding of the regrouping principle, which is particularly evident in the solutions on the worksheet. Here he consistently ignores regrouping in addition tasks, whereas in subtraction tasks he avoids decomposition by routinely subtracting the smaller digit from the larger one.

Furthermore, Child A demonstrates only a limited conceptual understanding of addition and subtraction. This is evident in the word problems, all of which he solves exclusively by applying a keyword-based strategy. Consequently, he solves all inconsistent word problems – those in which the relational language conflicts with the required operation – using an inappropriate arithmetic operation.

In addition to the work sheets, participants can access information on Child A’s language skills, and his achievements in German and science and social studies class. They can also view material that is less or not relevant to the case assessment, such as a sick note or a mandala he drew.

**Table S1. Overview of sample case A concerning the school performance and behaviour**

| Sample case “A” | |
| --- | --- |
| Dimension | Level of expression |
| Academic Performance | |
| German class | 3 |
| Mathematics class | 1 |
| Overall | 3 |
| Behaviour | |
| Hyperactivity | 4 |
| Impulsivity | 4 |
| Inattentiveness | 4 |
| Social functioning | 2 |
| Emotional self-regulation and frustration tolerance | 2 |
| Language Skills | |
| Speaking skills, vocabulary, and language comprehension | 3 |
| Writing skills (spelling, accuracy in grammar and punctuation, and written expression) | 3 |
| Reading skills (reading speed, reading accuracy, reading comprehension) | 2 |
| Mathematical Skills | |
| Place-value understanding | 2 |
| Magnitude representation of numbers | 1 |
| At least one transferable computation strategy | 1 |
| Conceptual understanding of addition/subtraction | 1 |
| Cognitive Skills | |
| Working memory & memory capacity | 3 |
| Cognitive flexibility, flexible task switching, logical reasoning | 2 |
| Processing speed (e.g., time needed to complete tasks, speed in routine tasks),  reaction and decision time | 3 |

*Note*. Levels of expression range from 1 = “very low” to 4 = “very high”. These levels are heuristic and designed to support the development of the fictive digital case scenarios. The boundaries between levels should be interpreted as approximate rather than discrete.
