# Supplementary material for: Enhancing diagnostic and intervention-related skills with adaptive feedback: Study protocol for a simulation-based learning study on symptoms of learning and behavioural difficulties
Source: PLoS One. 2026 Nov 13;21(11):e0361425. doi: 10.1371/journal.pone.0361425 (PMC13766221; doi:10.1371/journal.pone.0361425)
Supplement: Supplementary file 2 [file pone.0361425.s002.docx]

**No.:** PONE-D-26-26245

**Title:** Enhancing diagnostic and intervention-related skills with adaptive feedback: Study protocol for a simulation-based learning study on symptoms of learning and behavioural difficulties

Dear Editor,

We thank you and the reviewers for the careful consideration of our manuscript.

The comments provided were both insightful and constructive and have contributed to improving the quality of the manuscript. We have addressed each point in detail, with the corresponding modifications and additions highlighted in blue within the tracked changes version of the manuscript. The following tables summarise the comments and our responses, and we hope that these adequately address all concerns raised.

We are sincerely thankful for the time and effort invested in reviewing our work.

Best regards,
Corresponding author

**Editor Comments:**

|  | Comments | Responses |
| --- | --- | --- |
| 1. | Please ensure that your manuscript meets PLOS ONE's style requirements, including those for file naming. | Many thanks for the comment. We checked the manuscript against the guidelines and edited it accordingly. |
| 2. | Thank you for stating the following financial disclosure:  “This project is funded by the Deutsche Forschungsgemeinschaft (DFG, German Research Foundation) – TRR 419/1 – 542251251. The design of the study, collection, analysis and interpretation of data and writing the manuscript was not influenced by the DFG.”  Please state what role the funders took in the study.  If the funders had no role, please state: "The funders had no role in study design, data collection and analysis, decision to publish, or preparation of the manuscript."  If this statement is not correct you must amend it as needed.  Please include this amended Role of Funder statement in your cover letter; we will change the online submission form on your behalf. | We clarified the role the funders took in the study outline and the present manuscript and use the suggested wording:  The funders had no role in study design, data collection and analysis, decision to publish, or preparation of the manuscript.  This information can be found in the online submission system and we included it also in a separate cover letter. |
| 3. | Please note that funding information should not appear in any section or other areas of your manuscript. We will only publish funding information present in the Funding Statement section of the online submission form. Please remove any funding-related text from the manuscript. | Thanks for this information – we removed any funding information in the manuscript. |
| 4 | In the online submission form, you indicated that “The raw data supporting the conclusions of this manuscript will be made available by the authors, without undue reservation, to any qualified researcher on reasonable request.”  All PLOS journals now require all data underlying the findings described in their manuscript to be freely available to other researchers, either 1. In a public repository, 2. Within the manuscript itself, or 3. Uploaded as supplementary information.  This policy applies to all data except where public deposition would breach compliance with the protocol approved by your research ethics board. If your data cannot be made publicly available for ethical or legal reasons (e.g., public availability would compromise patient privacy), please explain your reasons on resubmission and your exemption request will be escalated for approval.  Additional Editor Comments:  Please specify the availability of the data. For example, anonymized research data, a data dictionary, analysis scripts, and materials used could be made available via OSF or Zenodo after the study is completed. If certain data cannot be published for ethical or data protection reasons, please provide specific justification and describe a regulated access procedure. | After careful consideration, we decided to update our data availability statement to conform to open science standards and to the requirements of PLOS journals:  The anonymised raw data supporting the conclusions of the studies described in this study protocol will be made available by the authors, through the public repository Zenodo. |
| 5. | Please include your full ethics statement in the ‘Methods’ section of your manuscript file. In your statement, please include the full name of the IRB or ethics committee who approved or waived your study, as well as whether or not you obtained informed written or verbal consent. If consent was waived for your study, please include this information in your statement as well. | Many thanks - the full ethics statement is included in our methods section:  For all research activities in this project, ethics is an integral part from beginning to end. Ethical evaluation and approval of the project was conducted and given by the ethics committee of the Faculty of Mathematics, Computer Science and Statistics, University of Munich (LMU; EK-MIS-2024-297).  All research actions in this project adhere to the ethical standards of data protection regulations according to Art. 13 EU-DSGVO. All participants in this project will be informed about these data regulations. Further, they will also be informed that their participation is voluntary and that all data will be pseudonymised and published findings will be anonymised. Only participants who understand this procedure and who gave their written consent to these regulations of data handling and processing will take part in this research. |
| 6. | If the reviewer comments include a recommendation to cite specific previously published works, please review and evaluate these publications to determine whether they are relevant and should be cited. There is no requirement to cite these works unless the editor has indicated otherwise. | There were no recommendations for additional literature suggested by the reviewers. |
| 7. | Please review your reference list to ensure that it is complete and correct. If you have cited papers that have been retracted, please include the rationale for doing so in the manuscript text, or remove these references and replace them with relevant current references. Any changes to the reference list should be mentioned in the rebuttal letter that accompanies your revised manuscript. If you need to cite a retracted article, indicate the article’s retracted status in the References list and also include a citation and full reference for the retraction notice. | The reference list was checked again, and no retractions of the cited articles were found. However, one duplicate entry was removed. |
| 8. | Additional Editor Comments:  Please document the LLM-based feedback in greater detail. This includes, for example, the provider and model name, model version or access date, system and user prompts, temperature and top-p settings, and quality control procedures. Significant changes to the model or prompts during the study should also be documented. | Many thanks for your comment. We added further details on the LLM-based feedback implementation in the manuscript. The changes are presented and discussed in our response to comment 2 of Reviewer #1 below. |

**Reviewer #1:**

|  | Comments | Responses |
| --- | --- | --- |
| 1. | The study protocol is acceptable following minor revisions. It addresses a relevant topic: simulation-based learning, adaptive feedback and the development of diagnostic and intervention-related skills in relation to learning and behavioural difficulties. The research rationale is clear, the hypotheses are well-founded, and the planned study design is, in principle, suitable for testing the research questions. The methodology is feasible and well-structured. The case vignettes, the CASUS environment and the three forms of feedback are described in a plausible manner. | Thank you very much for taking the time to review our study protocol and thank you for your constructive feedback and suggestions. |
| 2. | However, to ensure full replicability, the model version, prompting strategy, quality assurance of the LLM feedback (…) should be specified in greater detail. | At the present stage of the project, we only start with the testing of our feedback system with the model stated below, which is currently implemented in the CASUS learning platform. However, we added further details about our current and planned approach to implement and test the LLM feedback:  To generate this feedback, an API-based integration with a large language model (LLM; specifically, gpt-oss-120b, hosted locally on a university server) is used after learners submit their responses. The LLM is implemented with a standardised system prompt. The prompt includes instruction on the expected feedback content and structure, including a few-shot component that comprises a small number of feedback examples. In addition, to integrate sufficient information on the case content and adequate processing, the case-specific expert solution (similar to the static feedback) is provided as a reference material. Considering the rapid pace of technological developments in this field, the model may be replaced with a more up-to-date version in future iterations of the platform. […] The quality of the generated feedback will be established through testing, prior to data collection, whereby LLM outputs will be evaluated by subject-matter experts for accuracy, responsiveness to the individual learner’s submission, and alignment with the expert solution. |
| 3. | However, to ensure full replicability, the (…) exclusion criteria, dropout rules and handling of missing data should be specified in greater detail. | We added further information on exclusion criteria and dropout rules in the “Samples” section:  Participants are eligible for inclusion only if they belong to the relevant study groups for the respective study, individuals outside these groups are not recruited. For the intervention studies, only participants with complete study participation will be included in the analysis. Reasons for discontinuation or withdrawal from the study will be documented for all participants who do not complete the study.  Handling of missing data is now described in the “Analytic strategy” section:  Missing data within completed responses is minimised by the laboratory-based study setting, in which all questions are mandatory to complete before proceeding. However, this does not preclude participants from discontinuing their participation entirely, in which case the dropout will be documented and the participant’s data will be excluded. |
| 4. | The analysis plan is generally appropriate but still too general. Please specify the primary and secondary outcomes, planned comparisons, correction for multiple testing and the analysis of nested measurements. For three feedback conditions, a simple two-sample t-test is insufficient if static, macro-adaptive and micro-adaptive feedback are to be tested separately. | Following your suggestion, in the related section, we decided to update and extend our “Analysis plan” for the hypotheses concerning the three feedback conditions or study groups:  For the validation studies differences in prior knowledge and diagnostic- and intervention-related skills between the three student groups (teacher students, medical students, and school psychology students) will be examined using a one-way analysis of variance (ANOVA), with planned contrast analyses specified a priori to compare teacher students against medical and school psychology students, and medical students against teacher and school psychology students, according to hypotheses 1.1 & 1.2. Further, a one-way ANOVA with planned contrast analyses will be used to compare the three feedback conditions (static, macro-adaptive, and micro-adaptive) regarding the diagnostic- and intervention-related skills (hypothesis 1.3). |
| 5. | The data availability statement needs to be amended. ‘Available upon reasonable request’ is too vague for PLOS ONE. Please state that, upon completion of the study, data, materials, code, prompts and analysis scripts will be made available in a public repository, insofar as this is ethically and legally possible. | After careful consideration, we decided to update our data availability statement to conform to open science standards:  The anonymised raw data supporting the conclusions of the studies described in this study protocol will be made available by the authors, through the public repository Zenodo. |
| 6. | The language is generally clear. Please correct minor grammatical, typographical and formatting errors before acceptance. There are no obvious concerns regarding research ethics, publication ethics or duplicate publication | Many thanks for this comment. We checked the manuscript for any remaining language issues and edited it accordingly. |

**Reviewer #2:**

|  | Comments | Responses |
| --- | --- | --- |
| 5. | This is an exceptionally well-structured and timely study protocol. The proposed research addresses a highly relevant gap in higher education: training the diagnostic and intervention skills of future teachers, psychologists, and medical professionals using AI-driven, simulation-based learning. The rationale for comparing static, macro-adaptive, and NLP-powered micro-adaptive feedback is grounded in robust theoretical frameworks. The methodological design - spanning four sequential sub-studies—is rigorous, clearly detailing the sample sizes, power analyses, and variables of interest. Furthermore, the use of the CASUS platform with multimodal simulated case scenarios (e.g., worksheets, teacher reports) ensures high ecological validity. The ethical considerations and data availability statements are fully compliant with journal standards. This protocol provides a clear, highly promising roadmap for future empirical work. | Thank you very much for taking the time to review our study protocol and thank you for this very positive evaluation of our theoretical basis and study designs. |

**Reviewer #3:**

|  | Comments | Responses |
| --- | --- | --- |
| 6. | This study protocol outlines a promising, well-designed research project utilizing the CASUS simulation platform to train university students in diagnosing learning and behavioral difficulties. Its innovative approach comparing static, macro-adaptive, and NLP-driven micro-adaptive feedback across different student cohorts (medical, psychology, teaching) is highly relevant. The methodology is rigorous and clear. | Thank you very much for taking the time to review our study protocol and thank you for this very positive evaluation of our feedback comparison and our methodological approach. |
