# Supplementary material for: Enhancing diagnostic and intervention-related skills with adaptive feedback: Study protocol for a simulation-based learning study on symptoms of learning and behavioural difficulties
Source: PLoS One. 2026 Nov 13;21(11):e0361425. doi: 10.1371/journal.pone.0361425 (PMC13766221; doi:10.1371/journal.pone.0361425)
Supplement: Peer review history

# Peer review: Decision Letter 0

**DOI:** 10.1371/journal.pone.0361425.r001

**Authors:** Tobias Oberdieck

17 Aug 2026

Dear Dr. Niklas,

Thank you for submitting your manuscript to PLOS ONE. After careful consideration, we feel that it has merit but does not fully meet PLOS ONE’s publication criteria as it currently stands. Therefore, we invite you to submit a revised version of the manuscript that addresses the points raised during the review process.

Please submit your revised manuscript by Sep 01 2026 11:59PM. If you will need more time than this to complete your revisions, please reply to this message or contact the journal office at plosone@plos.org. When you're ready to submit your revision, log on to https://www.editorialmanager.com/pone/ and select the 'Submissions Needing Revision' folder to locate your manuscript file.

- A letter that responds to each point raised by the academic editor and reviewer(s). You should upload this letter as a separate file labeled 'Response to Reviewers'.
- A marked-up copy of your manuscript that highlights changes made to the original version. You should upload this as a separate file labeled 'Revised Manuscript with Track Changes'.
- An unmarked version of your revised paper without tracked changes. You should upload this as a separate file labeled 'Manuscript'.

If you would like to make changes to your financial disclosure, please include your updated statement in your cover letter. Guidelines for resubmitting your figure files are available below the reviewer comments at the end of this letter.

If applicable, we recommend that you deposit your laboratory protocols in protocols.io to enhance the reproducibility of your results. Protocols.io assigns your protocol its own identifier (DOI) so that it can be cited independently in the future. For instructions see: https://journals.plos.org/plosone/s/submission-guidelines#loc-laboratory-protocols. Additionally, PLOS ONE offers an option for publishing peer-reviewed Lab Protocol articles, which describe protocols hosted on protocols.io. Read more information on sharing protocols at https://plos.org/protocols?utm_medium=editorial-email&utm_source=authorletters&utm_campaign=protocols.

As the corresponding author, your ORCID iD is verified in the submission system and will appear in the published article. PLOS supports the use of ORCID, and we encourage all coauthors to register for an ORCID iD and use it as well. Please encourage your coauthors to verify their ORCID iD within the submission system before final acceptance, as unverified ORCID iDs will not appear in the published article. *Only*  the individual author can complete the verification step; PLOS staff *cannot*  verify ORCID iDs on behalf of authors.

We look forward to receiving your revised manuscript.

Kind regards,

Tobias Oberdieck, Dr., LL.M., B.A.

Academic Editor

PLOS One

Journal Requirements:

When submitting your revision, we need you to address these additional requirements.

1.Please ensure that your manuscript meets PLOS ONE's style requirements, including those for file naming. The PLOS ONE style templates can be found at

https://journals.plos.org/plosone/s/file?id=wjVg/PLOSOne_formatting_sample_main_body.pdf and

https://journals.plos.org/plosone/s/file?id=ba62/PLOSOne_formatting_sample_title_authors_affiliations.pdf

2. Thank you for stating the following financial disclosure:

“This project is funded by the Deutsche Forschungsgemeinschaft (DFG, German Research Foundation) – TRR 419/1 – 542251251. The design of the study, collection, analysis and interpretation of data and writing the manuscript was not influenced by the DFG.”

Please state what role the funders took in the study.  If the funders had no role, please state: "The funders had no role in study design, data collection and analysis, decision to publish, or preparation of the manuscript."

If this statement is not correct you must amend it as needed.

Please include this amended Role of Funder statement in your cover letter; we will change the online submission form on your behalf.

3. Please note that funding information should not appear in any section or other areas of your manuscript. We will only publish funding information present in the Funding Statement section of the online submission form. Please remove any funding-related text from the manuscript.

4. In the online submission form, you indicated that “The raw data supporting the conclusions of this manuscript will be made available by the authors, without undue reservation, to any qualified researcher on reasonable request.”

All PLOS journals now require all data underlying the findings described in their manuscript to be freely available to other researchers, either 1. In a public repository, 2. Within the manuscript itself, or 3. Uploaded as supplementary information.

This policy applies to all data except where public deposition would breach compliance with the protocol approved by your research ethics board. If your data cannot be made publicly available for ethical or legal reasons (e.g., public availability would compromise patient privacy), please explain your reasons on resubmission and your exemption request will be escalated for approval.

5. Please include your full ethics statement in the ‘Methods’ section of your manuscript file. In your statement, please include the full name of the IRB or ethics committee who approved or waived your study, as well as whether or not you obtained informed written or verbal consent. If consent was waived for your study, please include this information in your statement as well.

6. If the reviewer comments include a recommendation to cite specific previously published works, please review and evaluate these publications to determine whether they are relevant and should be cited. There is no requirement to cite these works unless the editor has indicated otherwise.

7. Please review your reference list to ensure that it is complete and correct. If you have cited papers that have been retracted, please include the rationale for doing so in the manuscript text, or remove these references and replace them with relevant current references. Any changes to the reference list should be mentioned in the rebuttal letter that accompanies your revised manuscript. If you need to cite a retracted article, indicate the article’s retracted status in the References list and also include a citation and full reference for the retraction notice.

Additional Editor Comments:

The study protocol addresses a relevant research question and is, overall, well-designed. However, several points should be clarified before the protocol can be accepted:

Please specify the availability of the data. For example, anonymized research data, a data dictionary, analysis scripts, and materials used could be made available via OSF or Zenodo after the study is completed. If certain data cannot be published for ethical or data protection reasons, please provide specific justification and describe a regulated access procedure.

Please document the LLM-based feedback in greater detail. This includes, for example, the provider and model name, model version or access date, system and user prompts, temperature and top-p settings, and quality control procedures. Significant changes to the model or prompts during the study should also be documented.

Following these minor revisions, I consider the manuscript ready for publication.

[Note: HTML markup is below. Please do not edit.]

Reviewer's Responses to Questions

**Comments to the Author**

1. Does the manuscript provide a valid rationale for the proposed study, with clearly identified and justified research questions?

Reviewer #1: Yes

Reviewer #2: Yes

Reviewer #3: Yes

**********

2. Is the protocol technically sound and planned in a manner that will lead to a meaningful outcome and allow testing the stated hypotheses?

Reviewer #1: Partly

Reviewer #2: Yes

Reviewer #3: Yes

**********

3. Is the methodology feasible and described in sufficient detail to allow the work to be replicable?

Reviewer #1: Yes

Reviewer #2: Yes

Reviewer #3: Yes

**********

4. Have the authors described where all data underlying the findings will be made available when the study is complete?

The PLOS Data policy

Reviewer #1: No

Reviewer #2: Yes

Reviewer #3: Yes

**********

5. Is the manuscript presented in an intelligible fashion and written in standard English?

Reviewer #1: Yes

Reviewer #2: Yes

Reviewer #3: Yes

**********

Please use the space provided to explain your answers to the questions above and, if applicable, provide comments about issues authors must address before this protocol can be accepted for publication. You may also include additional comments for the author, including concerns about research or publication ethics.

You may also provide optional suggestions and comments to authors that they might find helpful in planning their study.

Reviewer #1: The study protocol is acceptable following minor revisions. It addresses a relevant topic: simulation-based learning, adaptive feedback and the development of diagnostic and intervention-related skills in relation to learning and behavioural difficulties. The research rationale is clear, the hypotheses are well-founded, and the planned study design is, in principle, suitable for testing the research questions.

The methodology is feasible and well-structured. The case vignettes, the CASUS environment and the three forms of feedback are described in a plausible manner. However, to ensure full replicability, the model version, prompting strategy, quality assurance of the LLM feedback, data protection, exclusion criteria, dropout rules and handling of missing data should be specified in greater detail.

The analysis plan is generally appropriate but still too general. Please specify the primary and secondary outcomes, planned comparisons, correction for multiple testing and the analysis of nested measurements. For three feedback conditions, a simple two-sample t-test is insufficient if static, macro-adaptive and micro-adaptive feedback are to be tested separately.

The data availability statement needs to be amended. ‘Available upon reasonable request’ is too vague for PLOS ONE. Please state that, upon completion of the study, data, materials, code, prompts and analysis scripts will be made available in a public repository, insofar as this is ethically and legally possible.

The language is generally clear. Please correct minor grammatical, typographical and formatting errors before acceptance. There are no obvious concerns regarding research ethics, publication ethics or duplicate publication.

Reviewer #2: This is an exceptionally well-structured and timely study protocol. The proposed research addresses a highly relevant gap in higher education: training the diagnostic and intervention skills of future teachers, psychologists, and medical professionals using AI-driven, simulation-based learning.

The rationale for comparing static, macro-adaptive, and NLP-powered micro-adaptive feedback is grounded in robust theoretical frameworks. The methodological design - spanning four sequential sub-studies—is rigorous, clearly detailing the sample sizes, power analyses, and variables of interest.

Furthermore, the use of the CASUS platform with multimodal simulated case scenarios (e.g., worksheets, teacher reports) ensures high ecological validity. The ethical considerations and data availability statements are fully compliant with journal standards. This protocol provides a clear, highly promising roadmap for future empirical work.

Reviewer #3: This study protocol outlines a promising, well-designed research project utilizing the CASUS simulation platform to train university students in diagnosing learning and behavioral difficulties. Its innovative approach comparing static, macro-adaptive, and NLP-driven micro-adaptive feedback across different student cohorts (medical, psychology, teaching) is highly relevant. The methodology is rigorous and clear.

**********

what does this mean?). If published, this will include your full peer review and any attached files.

If you choose “no”, your identity will remain anonymous but your review may still be made public.

**Do you want your identity to be public for this peer review?**  For information about this choice, including consent withdrawal, please see our Privacy Policy

Reviewer #1: No

Reviewer #2: No

Reviewer #3: No

**********

[NOTE: If reviewer comments were submitted as an attachment file, they will be attached to this email and accessible via the submission site. Please log into your account, locate the manuscript record, and check for the action link "View Attachments". If this link does not appear, there are no attachment files.]

To ensure your figures meet our technical requirements, please review our figure guidelines: https://journals.plos.org/plosone/s/figures

You may also use PLOS’s free figure tool, NAAS, to help you prepare publication quality figures: https://journals.plos.org/plosone/s/figures#loc-tools-for-figure-preparation.

NAAS will assess whether your figures meet our technical requirements by comparing each figure against our figure specifications.

# Author comment: Author response to Decision Letter 1

**DOI:** 10.1371/journal.pone.0361425.r002

11 Sep 2026

No.: PONE-D-26-26245

Title: Enhancing diagnostic and intervention-related skills with adaptive feedback: Study protocol for a simulation-based learning study on symptoms of learning and behavioural difficulties

Dear Editor,

We thank you and the reviewers for the careful consideration of our manuscript.

The comments provided were both insightful and constructive and have contributed to improving the quality of the manuscript. We have addressed each point in detail, with the corresponding modifications and additions highlighted in blue within the tracked changes version of the manuscript. The following tables summarise the comments and our responses, and we hope that these adequately address all concerns raised.

We are sincerely thankful for the time and effort invested in reviewing our work.

Best regards,

Corresponding author

Editor Comments:

Comments & Responses

1. Please ensure that your manuscript meets PLOS ONE's style requirements, including those for file naming.

-> Many thanks for the comment. We checked the manuscript against the guidelines and edited it accordingly.

2. Thank you for stating the following financial disclosure:

“This project is funded by the Deutsche Forschungsgemeinschaft (DFG, German Research Foundation) – TRR 419/1 – 542251251. The design of the study, collection, analysis and interpretation of data and writing the manuscript was not influenced by the DFG.”

Please state what role the funders took in the study. If the funders had no role, please state: "The funders had no role in study design, data collection and analysis, decision to publish, or preparation of the manuscript."

If this statement is not correct you must amend it as needed.

Please include this amended Role of Funder statement in your cover letter; we will change the online submission form on your behalf.

-> We clarified the role the funders took in the study outline and the present manuscript and use the suggested wording:

The funders had no role in study design, data collection and analysis, decision to publish, or preparation of the manuscript.

This information can be found in the online submission system and we included it also in a separate cover letter.

3. Please note that funding information should not appear in any section or other areas of your manuscript. We will only publish funding information present in the Funding Statement section of the online submission form. Please remove any funding-related text from the manuscript.

-> Thanks for this information – we removed any funding information in the manuscript.

4 In the online submission form, you indicated that “The raw data supporting the conclusions of this manuscript will be made available by the authors, without undue reservation, to any qualified researcher on reasonable request.”

All PLOS journals now require all data underlying the findings described in their manuscript to be freely available to other researchers, either 1. In a public repository, 2. Within the manuscript itself, or 3. Uploaded as supplementary information.

This policy applies to all data except where public deposition would breach compliance with the protocol approved by your research ethics board. If your data cannot be made publicly available for ethical or legal reasons (e.g., public availability would compromise patient privacy), please explain your reasons on resubmission and your exemption request will be escalated for approval.

Additional Editor Comments:

Please specify the availability of the data. For example, anonymized research data, a data dictionary, analysis scripts, and materials used could be made available via OSF or Zenodo after the study is completed. If certain data cannot be published for ethical or data protection reasons, please provide specific justification and describe a regulated access procedure.

-> After careful consideration, we decided to update our data availability statement to conform to open science standards and to the requirements of PLOS journals:

"The anonymised raw data supporting the conclusions of the studies described in this study protocol will be made available by the authors, through the public repository Zenodo."

5. Please include your full ethics statement in the ‘Methods’ section of your manuscript file. In your statement, please include the full name of the IRB or ethics committee who approved or waived your study, as well as whether or not you obtained informed written or verbal consent. If consent was waived for your study, please include this information in your statement as well.

-> Many thanks - the full ethics statement is included in our methods section:

For all research activities in this project, ethics is an integral part from beginning to end. Ethical evaluation and approval of the project was conducted and given by the ethics committee of the Faculty of Mathematics, Computer Science and Statistics, University of Munich (LMU; EK-MIS-2024-297).

All research actions in this project adhere to the ethical standards of data protection regulations according to Art. 13 EU-DSGVO. All participants in this project will be informed about these data regulations. Further, they will also be informed that their participation is voluntary and that all data will be pseudonymised and published findings will be anonymised. Only participants who understand this procedure and who gave their written consent to these regulations of data handling and processing will take part in this research.

6. If the reviewer comments include a recommendation to cite specific previously published works, please review and evaluate these publications to determine whether they are relevant and should be cited. There is no requirement to cite these works unless the editor has indicated otherwise.

-> There were no recommendations for additional literature suggested by the reviewers.

7. Please review your reference list to ensure that it is complete and correct. If you have cited papers that have been retracted, please include the rationale for doing so in the manuscript text, or remove these references and replace them with relevant current references. Any changes to the reference list should be mentioned in the rebuttal letter that accompanies your revised manuscript. If you need to cite a retracted article, indicate the article’s retracted status in the References list and also include a citation and full reference for the retraction notice.

-> The reference list was checked again, and no retractions of the cited articles were found. However, one duplicate entry was removed.

8. Additional Editor Comments:

Please document the LLM-based feedback in greater detail. This includes, for example, the provider and model name, model version or access date, system and user prompts, temperature and top-p settings, and quality control procedures. Significant changes to the model or prompts during the study should also be documented.

-> Many thanks for your comment. We added further details on the LLM-based feedback implementation in the manuscript. The changes are presented and discussed in our response to comment 2 of Reviewer #1 below.

Reviewer #1:

Comments & Responses

1. The study protocol is acceptable following minor revisions. It addresses a relevant topic: simulation-based learning, adaptive feedback and the development of diagnostic and intervention-related skills in relation to learning and behavioural difficulties. The research rationale is clear, the hypotheses are well-founded, and the planned study design is, in principle, suitable for testing the research questions.

The methodology is feasible and well-structured. The case vignettes, the CASUS environment and the three forms of feedback are described in a plausible manner.

-> Thank you very much for taking the time to review our study protocol and thank you for your constructive feedback and suggestions.

2. However, to ensure full replicability, the model version, prompting strategy, quality assurance of the LLM feedback (…) should be specified in greater detail.

-> At the present stage of the project, we only start with the testing of our feedback system with the model stated below, which is currently implemented in the CASUS learning platform. However, we added further details about our current and planned approach to implement and test the LLM feedback:

To generate this feedback, an API-based integration with a large language model (LLM; specifically, gpt-oss-120b, hosted locally on a university server) is used after learners submit their responses. The LLM is implemented with a standardised system prompt. The prompt includes instruction on the expected feedback content and structure, including a few-shot component that comprises a small number of feedback examples. In addition, to integrate sufficient information on the case content and adequate processing, the case-specific expert solution (similar to the static feedback) is provided as a reference material. Considering the rapid pace of technological developments in this field, the model may be replaced with a more up-to-date version in future iterations of the platform. […] The quality of the generated feedback will be established through testing, prior to data collection, whereby LLM outputs will be evaluated by subject-matter experts for accuracy, responsiveness to the individual learner’s submission, and alignment with the expert solution.

3. However, to ensure full replicability, the (…) exclusion criteria, dropout rules and handling of missing data should be specified in greater detail.

-> We added further information on exclusion criteria and dropout rules in the “Samples” section:

Participants are eligible for inclusion only if they belong to the relevant study groups for the respective study, individuals outside these groups are not recruited. For the intervention studies, only participants with complete study participation will be included in the analysis. Reasons for discontinuation or withdrawal from the study will be documented for all participants who do not complete the study.

Handling of missing data is now described in the “Analytic strategy” section:

Missing data within completed responses is minimised by the laboratory-based study setting, in which all questions are mandatory to complete before proceeding. However, this does not preclude participants from discontinuing their participation entirely, in which case the dropout will be documented and the participant’s data will be excluded.

4. The analysis plan is generally appropriate but still too general. Please specify the primary and secondary outcomes, planned comparisons, correction for multiple testing and the analysis of nested measurements. For three feedback conditions, a simple two-sample t-test is insufficient if static, macro-adaptive and micro-adaptive feedback are to be tested separately.

-> Following your suggestion, in the related section, we decided to update and extend our “Analysis plan” for the hypotheses concerning the three feedback conditions or study groups:

For the validation studies differences in prior knowledge and diagnostic- and intervention-related skills between the three student groups (teacher students, medical students, and school psychology students) will be examined using a one-way analysis of variance (ANOVA), with planned contrast analyses specified a priori to compare teacher students against medical and school psychology students, and medical students against teacher and school psychology students, according to hypotheses 1.1 & 1.2. Further, a one-way ANOVA with planned contrast analyses will be used to compare the three feedback conditions (static, macro-adaptive, and micro-adaptive) regarding the diagnostic- and intervention-related skills (hypothesis 1.3).

5. The data availability statement needs to be amended. ‘Available upon reasonable request’ is too vague for PLOS ONE. Please state that, upon completion of the study, data, materials, code, prompts and analysis scripts will be made available in a public repository, insofar as this is ethically and legally possible.

-> After careful consideration, we decided to update our data availability statement to conform to open science standards:

The anonymised raw data supporting the conclusions of the studies described in this study protocol will be made available by the authors, through the public repository Zenodo.

6. The language is generally clear. Please correct minor grammatical, typographical and formatting errors before acceptance. There are no obvious concerns regarding research ethics, publication ethics or duplicate publication

-> Many thanks for this comment. We checked the manuscript for any remaining language issues and edited it accordingly.

Reviewer #2:

Comments & Responses

5. This is an exceptionally well-structured and timely study protocol. The proposed research addresses a highly relevant gap in higher education: training the diagnostic and intervention skills of future teachers, psychologists, and medical professionals using AI-driven, simulation-based learning.

The rationale for comparing static, macro-adaptive, and NLP-powered micro-adaptive feedback is grounded in robust theoretical frameworks. The methodological design - spanning four sequential sub-studies—is rigorous, clearly detailing the sample sizes, power analyses, and variables of interest.

Furthermore, the use of the CASUS platform with multimodal simulated case scenarios (e.g., worksheets, teacher reports) ensures high ecological validity. The ethical considerations and data availability statements are fully compliant with journal standards. This protocol provides a clear, highly promising roadmap for future empirical work.

-> Thank you very much for taking the time to review our study protocol and thank you for this very positive evaluation of our theoretical basis and study designs.

Reviewer #3:

Comments & Responses

This study protocol outlines a promising, well-designed research project utilizing the CASUS simulation platform to train university students in diagnosing learning and behavioral difficulties. Its innovative approach comparing static, macro-adaptive, and NLP-driven micro-adaptive feedback across different student cohorts (medical, psychology, teaching) is highly relevant. The methodology is rigorous and clear.

-> Thank you very much for taking the time to review our study protocol and thank you for this very positive evaluation of our feedback comparison and our methodological approach.

Attachment Submitted filename: Response to Reviewers.docx

# Peer review: Decision Letter 1

**DOI:** 10.1371/journal.pone.0361425.r003

**Authors:** Tobias Oberdieck

12 Oct 2026

Enhancing diagnostic and intervention-related skills with adaptive feedback: Study protocol for a simulation-based learning study on symptoms of learning and behavioural difficulties

PONE-D-26-26245R1

Dear Dr. Niklas,

We’re pleased to inform you that your manuscript has been judged scientifically suitable for publication and will be formally accepted for publication once it meets all outstanding technical requirements.

Within one week, you’ll receive an e-mail detailing the required amendments. When these have been addressed, you’ll receive a formal acceptance letter and your manuscript will be scheduled for publication.

An invoice will be generated when your article is formally accepted. Please note, if your institution has a publishing partnership with PLOS and your article meets the relevant criteria, all or part of your publication costs will be covered. Please make sure your user information is up-to-date by logging into Editorial Manager at Editorial Manager® and clicking the ‘Update My Information' link at the top of the page. For questions related to billing, please contact billing support.

If your institution or institutions have a press office, please notify them about your upcoming paper to help maximize its impact. If they’ll be preparing press materials, please inform our press team as soon as possible -- no later than 48 hours after receiving the formal acceptance. Your manuscript will remain under strict press embargo until 2 pm Eastern Time on the date of publication. For more information, please contact onepress@plos.org.

Kind regards,

Tobias Oberdieck, Dr., LL.M., B.A.

Academic Editor

PLOS One

Additional Editor Comments (optional):

Dear Authors,

Thank you very much for revising your manuscript and responding to the reviewers’ comments.

Having reviewed the revised version and taken the updated reviews into account, I am pleased to inform you that your study protocol has been accepted.

The additional information provided on the implementation of LLM-based feedback, the analysis plan and the provision of anonymised research data has improved the transparency of your work. Please treat the remaining comments from Reviewer 1 as recommendations for the conduct, documentation and subsequent reporting of your studies.

Thank you very much for your contribution and your constructive cooperation during the peer-review process.

Reviewers' comments:

Reviewer's Responses to Questions

**Comments to the Author**

1. Does the manuscript provide a valid rationale for the proposed study, with clearly identified and justified research questions?

Reviewer #1: Yes

Reviewer #2: Yes

Reviewer #3: Yes

**********

2. Is the protocol technically sound and planned in a manner that will lead to a meaningful outcome and allow testing the stated hypotheses?

Reviewer #1: Yes

Reviewer #2: Yes

Reviewer #3: Yes

**********

3. Is the methodology feasible and described in sufficient detail to allow the work to be replicable?

Reviewer #1: No

Reviewer #2: Yes

Reviewer #3: Yes

**********

4. Have the authors described where all data underlying the findings will be made available when the study is complete?

The PLOS Data policy

Reviewer #1: Yes

Reviewer #2: Yes

Reviewer #3: Yes

**********

5. Is the manuscript presented in an intelligible fashion and written in standard English?

Reviewer #1: Yes

Reviewer #2: Yes

Reviewer #3: Yes

**********

Please use the space provided to explain your answers to the questions above and, if applicable, provide comments about issues authors must address before this protocol can be accepted for publication. You may also include additional comments for the author, including concerns about research or publication ethics.

You may also provide optional suggestions and comments to authors that they might find helpful in planning their study.

Reviewer #1: The revised version has convincingly addressed the key points raised in the previous review. The study design is well structured and logically coherent, the hypotheses are clearly formulated, and the three feedback conditions are sufficiently distinguished. Further strengths include the reported power analyses, the detailed description of the samples and simulated cases, and the additional information on LLM-based feedback generation.

A few points should be clarified before final acceptance. First, the documentation of the LLM should be made fully reproducible. In addition to the model name, the model version or access date, system and user prompts, temperature and top-p settings, as well as procedures for documenting possible model changes, should be clearly specified. Second, the analysis plan for Studies 2–4 should define the primary and secondary outcomes more precisely, explain how multiple testing will be addressed, and describe in greater detail how repeated or nested measurements will be analysed. Third, Table 1 should be clarified, as some entries refer to a “2 group comparison” although three student groups or three feedback conditions are involved. Fourth, the data availability statement should, where possible, also include study materials, analysis scripts, code, and the prompts used for the LLM. Finally, the manuscript would benefit from a final proofreading.

Reviewer #2: This study protocol outlines a timely and well-designed research project using the CASUS simulation platform to train university students in diagnosing learning and behavioral difficulties. The authors have effectively addressed all reviewer comments. Crucially, they provided necessary details on the LLM-based micro-adaptive feedback implementation, clarified their handling of missing data, and refined the statistical analysis plan. The updated data availability statement aligns with open science standards. The rationale for comparing static, macro-adaptive, and micro-adaptive feedback is grounded in robust theoretical frameworks, and the methodological design is rigorous. The planned research offers a highly promising roadmap. Recommend accepting.

Reviewer #3: This manuscript presents a well-crafted and highly relevant experimental plan that leverages the CASUS environment to educate higher-education learners on identifying behavioral and cognitive challenges. The researchers successfully resolved every issue raised during peer review. Most importantly, they expanded on the technical specifics of their AI-driven personalized feedback, explained their approach to incomplete responses, and sharpened their quantitative assessment strategy. Furthermore, their revised data sharing policy fully complies with modern transparency norms. By building upon solid academic theories to contrast fixed, group-level, and highly individualized feedback types, this thorough experimental blueprint paves the way for impactful future investigations.

**********

what does this mean?). If published, this will include your full peer review and any attached files.

If you choose “no”, your identity will remain anonymous but your review may still be made public.

**Do you want your identity to be public for this peer review?**  For information about this choice, including consent withdrawal, please see our Privacy Policy

Reviewer #1: No

Reviewer #2: No

Reviewer #3: No

**********

# Editor report: Acceptance letter

**DOI:** 10.1371/journal.pone.0361425.r004

**Authors:** Tobias Oberdieck

PONE-D-26-26245R1

PLOS One

Dear Dr. Niklas,

I'm pleased to inform you that your manuscript has been deemed suitable for publication in PLOS One. Congratulations! Your manuscript is now being handed over to our production team.

At this stage, our production department will prepare your paper for publication. This includes ensuring the following:

* All references, tables, and figures are properly cited

* All relevant supporting information is included in the manuscript submission,

* There are no issues that prevent the paper from being properly typeset

You will receive further instructions from the production team, including instructions on how to review your proof when it is ready. Please keep in mind that we are working through a large volume of accepted articles, so please give us a few days to review your paper and let you know the next and final steps.

Lastly, if your institution or institutions have a press office, please let them know about your upcoming paper now to help maximize its impact. If they'll be preparing press materials, please inform our press team within the next 48 hours. Your manuscript will remain under strict press embargo until 2 pm Eastern Time on the date of publication. For more information, please contact onepress@plos.org.

You will receive an invoice from PLOS for your publication fee after your manuscript has reached the completed accept phase. If you receive an email requesting payment before acceptance or for any other service, this may be a phishing scheme. Learn how to identify phishing emails and protect your accounts at https://explore.plos.org/phishing.

If we can help with anything else, please email us at customercare@plos.org.

Thank you for submitting your work to PLOS One and supporting open access.

Kind regards,

PLOS One Editorial Office Staff

on behalf of

Dr. Tobias Oberdieck

Academic Editor

PLOS One
